# Explained and Residual Variability of the Fetal Mechanical PR Interval in Structurally Normal Hearts: A Secondary Analysis of Physiological, Biometric, and Doppler Covariates

**DOI:** 10.3390/biomedicines14102303

**Published:** 2026-10-10

**Authors:** Jakub Mlodawski, Katarzyna Janiak, Lukasz Pawlik, Marta Mlodawska, Jay Mehta, Grzegorz Swiercz

**Affiliations:** 1 Collegium Medicum, Jan Kochanowski University in Kielce, 25-369 Kielce, Poland; marta.mlodawska@ujk.edu.pl (M.M.); grzegorz.swiercz@ujk.edu.pl (G.S.); 2 Polish Mother’s Memorial Hospital Research Institute, 93-338 Lodz, Poland; kasiajaniak@me.com; 3 Department of Information Systems, Kielce University of Technology, 25-314 Kielce, Poland; lpawlik@tu.kielce.pl; 4 Shree IVF end Endometriosis Clinic, Mumbai 400077, India; shreeivfclinic@gmail.com

**Keywords:** fetal echocardiography, mechanical PR interval, fetal atrioventricular conduction, fetal heart rate, dominance analysis

## Abstract

**Background/Objectives:** A previous publication reported that routinely measured covariates explained approximately 10% of fetal mechanical PR interval (mPR) variance. This secondary analysis examined how explanatory information is distributed among correlated predictors, their incremental contributions beyond fetal heart rate (FHR), and performance across alternative model specifications. **Methods:** We analysed 903 complete cases with normal fetal echocardiographic findings. Predictors comprised FHR, gestational age (GA), biparietal diameter (BPD), fronto-occipital diameter (FOD), pulmonary valve diameter (PVD), and umbilical artery pulsatility index (UA PI). Hierarchical ordinary least squares regression was complemented by general dominance analysis across all 720 predictor entry orders, bootstrap uncertainty estimates, and sensitivity analyses using shared ten-fold outer cross-validation splits. **Results:** The full-model apparent R^2^ of 0.0986 was consistent with the previously reported low explanatory capacity. The additional analyses showed that FHR accounted for 51.23% of full-model explained variance in general dominance analysis. Adding GA after FHR increased R^2^ by 0.0237; subsequent additions of BPD, FOD and PVD, and then UA PI, increased R^2^ by 0.0061 and 0.0037, respectively (*p* = 0.112 and *p* = 0.057). Full-model pooled out-of-fold R^2^ was 0.0826. Exploratory gradient boosting increased apparent R^2^ to 0.1722 but yielded a lower out-of-fold R^2^ of 0.0554. **Conclusions:** This secondary analysis extends the previous report by quantifying incremental and shared explanatory information and comparing apparent fit with internal validation performance. FHR accounted for the largest average contribution across predictor entry orders, while the additional biometric and Doppler blocks provided limited incremental information after FHR and GA. The evaluated alternative specifications did not substantially improve internal validation performance. The low overall explained variance is consistent with the previous publication and is not a new finding. Model-unexplained variance cannot be attributed specifically to intrinsic atrioventricular conduction properties, because its biological and measurement-related components cannot be distinguished using the available data.

## 1. Introduction

The fetal mechanical PR interval (mPR) is used in fetal echocardiography as an ultrasonographic correlate of atrioventricular conduction. It is most commonly applied in the surveillance of fetuses of mothers with anti-SSA/Ro and anti-SSB/La antibodies, who are at risk of atrioventricular conduction abnormalities. Ref. [1] Measurement of mPR is widely accessible, as it can be performed using pulsed-wave Doppler in the mitral valve/aortic outflow tract (MV/Ao) view. In contrast, alternative methods allowing direct or near-direct assessment of fetal cardiac electrical activity, such as non-invasive fetal electrocardiography or fetal magnetocardiography, require specialized equipment and are not routinely available in outpatient clinical practice [2].

However, interpretation of mPR is not entirely straightforward. This parameter is not a direct electrical measurement, but rather a mechanical surrogate of atrioventricular conduction. Therefore, it may be influenced by fetal heart rate, gestational age, fetal size, biometric parameters, the dimensions of cardiac structures, Doppler indices, fetal behavioral state, and technical factors related to the measurement itself.

In a previous analysis of the same clinical source cohort, using an earlier analytical database, ref. [3] fetal heart rate was shown to be the strongest single predictor of mPR, whereas biometric parameters and the umbilical artery pulsatility index contributed only limited additional information to the modelling of mPR values. At the same time, the vast majority of the total variability in mPR remained unexplained by the included predictors. This raises an important interpretative question: is mPR largely determined by routinely measured physiological, biometric, and Doppler parameters, or does it retain substantial residual variability, potentially related to more complex regulation of atrioventricular conduction, biological variability, fetal behavioral state, or technical aspects of measurement?

## 2. Aim

This secondary analysis aimed to extend the previous report by quantifying the incremental contribution of successive predictor blocks, allocating shared explanatory information among correlated covariates using general dominance analysis, and comparing apparent fit with internal validation performance across model specifications. The previously reported low overall R^2^ and its complementary unexplained proportion are treated as background findings rather than new results. Ref. [3] the present study provides additional methodological and quantitative information, rather than independent replication; Table S1 distinguishes previously reported findings from the additional analyses.

## 3. Study Design and Data Source

The present study is a secondary analysis of data from the same clinical source cohort as the previous publication. The analytical databases were assembled at different stages of data aggregation. When the previous project was finalized, some eligible examinations had not yet been incorporated into the database, and that publication reported 925 eligible fetuses. Subsequent aggregation of additional eligible examinations yielded the 1004 source records available for the present analysis. Unlike the previous study, which described median imputation of missing values, the present analysis required complete data for mPR and all six candidate predictors. Of the 1004 records, 101 (10.1%) lacked at least one required measurement, leaving 903 complete cases. The analytical samples therefore were not identical. Fetuses were examined at the Fetal Cardiology Outpatient Clinic of the Provincial Integrated Hospital in Kielce between January 2023 and December 2024.

The original cohort included only fetuses with normal cardiac anatomy and function and no extracardiac malformations. Multiple pregnancies, cases with confirmed chromosomal aberrations or a positive NIPT result, patients receiving medications that could affect conduction within the cardiac conduction system, and pregnant women referred because of the presence of anti-SSA/Ro or anti-SSB/La antibodies were excluded.

## 4. Measurement of the Dependent Variable

The dependent variable was the fetal mechanical PR interval (mPR), expressed in milliseconds. The measurement was performed using pulsed-wave Doppler with simultaneous visualization of mitral inflow and aortic outflow in the MV/Ao view, corresponding to the five-chamber view of the fetal heart. Mechanical atrioventricular conduction time was defined as the interval from the onset of the mitral inflow A-wave to the onset of aortic outflow.

All measurements were performed by a single experienced sonographer certified in fetal echocardiography and with more than 20 years of clinical experience. The analytical dataset contained one recorded mPR value per fetus and no repeated measurements suitable for estimating intraobserver repeatability. Consequently, neither intraobserver repeatability nor interobserver reproducibility could be assessed. Use of a single operator avoided between-operator differences within the dataset but did not eliminate measurement error.

## 5. Analytical Sample and Variables

The analysis was conducted using a complete-case approach and included 903 observations with complete data for all variables entered into the models. The dependent variable was mPR expressed in milliseconds. Missingness was summarized for each variable and as mutually exclusive combinations of missing variables. Included and excluded records were compared descriptively using all available observations for each variable, with variable-specific denominators, means and standard deviations, medians and interquartile ranges, and standardized mean differences. No imputation was performed. Reasons for individual missing values were not recorded in the analytical dataset and could not be determined from the missingness patterns. Detailed results are provided in Table S7.

Gestational age was established from the last menstrual period. When menstrual dating differed from ultrasound dating at the first-trimester prenatal examination by more than 7 days, the first-trimester ultrasound estimate was used. Gestational age was not derived from fetal biometry obtained at the echocardiographic examination analysed in this study.

The predefined set of candidate predictors included gestational age (GA), biparietal diameter (BPD), fronto-occipital diameter (FOD), fetal heart rate (FHR), umbilical artery pulsatility index (UA PI), and pulmonary valve diameter (PVD). The selection of predictors was based on previous analyses of this cohort and on their potential physiological relevance.

FHR represented the current chronotropic state of the fetus. GA and biometric parameters reflected fetal maturation and growth. PVD served as an approximate marker of cardiac structure size, whereas UA PI represented a basic measure of resistance in the placental circulation.

## 6. Statistical Analysis

The statistical analysis was designed to evaluate the proportion of mPR variance explained by routinely recorded covariates, rather than solely to identify statistically significant predictors. Continuous variables were described using the mean and standard deviation, as well as the median and interquartile range, depending on the distributional characteristics and presentation requirements. Associations between mPR and candidate predictors, as well as correlations among the predictors themselves, were assessed using Spearman’s rank correlation coefficient.

Collinearity was evaluated using the correlation matrix, variance inflation factors (VIFs), and the condition number of the design matrix. Sequential ordinary least squares linear regression models were then constructed to estimate the incremental proportion of explained variance after the addition of successive predictor blocks. Model 1 included FHR only. Model 2 was extended by adding GA. Model 3 additionally included parameters reflecting fetal growth and cardiac size, represented by BPD, FOD, and PVD. Model 4 was further extended by adding UA PI.

The hierarchical sequence was used to assess whether gestational age, fetal and cardiac size, and UA PI provided additional explanatory information beyond FHR. This ordering addresses a conditional question rather than providing an order-independent ranking of predictor importance. We therefore complemented the hierarchical models with general dominance analysis. Each predictor’s incremental R^2^ was averaged across all 720 possible entry orders, using the same 903 complete cases for all subset models. General dominance weights sum to the full-model R^2^, and their relative shares were calculated by dividing each weight by that R^2^. We also calculated each predictor’s unique contribution as the reduction in R^2^ after its removal from the full model [4].

For each model, the coefficient of determination (R^2^), adjusted R^2^, the increment in R^2^ relative to the preceding model, RMSE, and the proportion of variance unexplained by the model were reported. The unexplained proportion of variance was calculated as (1 − R^2^) × 100. Improvements in model fit between nested models were assessed using the F-test.

Approximate 95% confidence intervals for apparent R^2^, adjusted R^2^, RMSE, partial R^2^ and incremental R^2^ were estimated using 5000 pair-bootstrap resamples of the 903 complete records, with the same resampled indices used for all model comparisons. Percentile intervals were calculated conditional on the specified models and did not account for prior model selection. Their limitations near zero, particularly for partial and incremental R^2^, are described in the Supplementary Methods. Results are presented in Table S6.

Sensitivity analyses included OLS with log-transformed mPR, OLS excluding GA, LASSO, ridge regression and exploratory gradient boosting. Apparent fit and internal validation performance were reported separately. The exploratory gradient-boosting model used 100 trees, a learning rate of 0.05, a maximum tree depth of 2, and a minimum of 10 observations per leaf; these settings were fixed without hyperparameter tuning. All models used the same shuffled ten-fold outer cross-validation splits. For LASSO and ridge, standardization and penalty selection were performed within the training data using an inner five-fold cross-validation procedure. Log-model predictions were retransformed to milliseconds using a training-set smearing factor. Detailed settings and performance metrics are provided in the Supplementary Methods and Table S3.

For the full and exploratory three-predictor models, diagnostic assessment included residual-versus-fitted, normal Q–Q and scale–location plots, the Koenker–Breusch–Pagan test, Shapiro–Wilk tests, and Ramsey RESET tests. Leverage and Cook’s distance were examined to identify potentially influential observations. Conventional inference was supplemented with HC3 heteroskedasticity-consistent standard errors, confidence intervals and Wald tests. Detailed procedures and results are provided in the Supplementary Methods, Table S5 and Figures S1 and S2.

The three-predictor model was evaluated as an exploratory reduced specification informed by the observed correlation structure and model comparisons. FHR represented heart rate, PVD provided a single measure of cardiac size from the correlated maturation and size variables, and UA PI represented placental Doppler status. UA PI was retained for domain representation rather than because it met a significance threshold. A two-predictor model excluding UA PI was also evaluated to quantify its incremental contribution (Table S4). The reduced specification was not treated as a prespecified or independently validated model, and it was not the exact predictor set selected by LASSO. For the exploratory reduced model, standardized β coefficients were calculated after z-score standardization of both the predictors and the dependent variable. Partial R^2^ values for individual predictors and VIFs were also calculated. All tests were two-sided, and the threshold for statistical significance was set at *p* < 0.05.

## 7. Software

All secondary analyses were performed in Python 3.11, ref. [5] using the pandas 2.2.3, NumPy 2.3.5, ref. [6] SciPy, ref. [7] statsmodels, ref. [8] scikit-learn, ref. [9] and matplotlib [10] libraries. The analyses performed during revision were conducted using Python 3.12.14; library versions are provided in the Supplementary Methods.

## 8. Results

Of the 1004 records in the source dataset, 903 (89.9%) had complete data for mPR and all six candidate predictors and were included in the analysis. Gestational age ranged from 15 to 38 weeks, with a median of 26 weeks (IQR, 25–29) and a mean of 26.9 ± 3.6 weeks. The median mPR was 116 ms (IQR: 107–123), with a mean of 115.7 ± 10.6 ms. The median FHR was 143 beats/min (IQR: 137–150). Detailed characteristics of the analytical cohort are presented in Table 1. Among the 1004 source records, 101 (10.1%) were excluded because at least one required variable was missing. UA PI was the most frequently missing variable (74 records), followed by PVD (22 records), mPR (18), FHR (17), FOD (5), BPD (3) and GA (2); these counts overlap. Missing UA PI alone accounted for 64 exclusions. Available mPR and FHR measurements were similar between included and excluded records. However, mean UA PI was higher among excluded records with an available measurement (1.29 ± 0.47; *n* = 27) than among included records (1.08 ± 0.22; *n* = 903). Variable-specific missingness, all observed missingness patterns and descriptive comparisons are presented in Table S7.

## 9. Correlation Structure and Multicollinearity Among Candidate Predictors

Correlation analysis (Table 2) showed that variables describing fetal maturation and growth formed a strongly correlated block of predictors. The highest correlation coefficients were observed among GA, BPD, and FOD, and positive associations were also found between these parameters and PVD.

With respect to the outcome variable, the strongest association was observed between mPR and FHR, whereas correlations between mPR and the remaining predictors were weak. Collinearity diagnostics confirmed substantial redundancy among variables reflecting fetal growth and maturation. High VIF values for GA, BPD, and FOD indicated that their simultaneous inclusion in the model could lead to instability of regression coefficient estimates.

For this reason, the subsequent analyses focused not only on the full model, but also on simplified models and alternative specifications aimed at reducing collinearity.

### 9.1. Incremental Explained Variance Across Hierarchical Models

To systematically assess the extent to which successive groups of variables improved the explanation of mPR variability, a hierarchical approach based on sequential expansion of regression models was applied. This analysis allowed estimation of the incremental proportion of explained variance after adding successive blocks of predictors and assessment of the magnitude of the mPR variability component that remained unexplained despite adjustment for accompanying physiological, biometric, and Doppler parameters. Detailed results are presented in Table 3.

Hierarchical regression modelling showed that FHR made the largest contribution to the model-explained variance in mPR. The model including FHR alone explained 6.5% of the total variability in mPR. Adding GA increased R^2^ to 0.089, corresponding to an additional 2.4 percentage points of explained variance. Inclusion of the fetal growth and cardiac size block, comprising BPD, FOD, and PVD, increased R^2^ only to 0.095. Further addition of UA PI resulted in a final R^2^ of 0.099. Neither the addition of the fetal growth and cardiac size block nor the subsequent addition of UA PI reached the conventional significance threshold in the hierarchical comparisons (*p* = 0.112 and *p* = 0.057, respectively; Table 3). The full model had an apparent R^2^ of 0.0986 (95% bootstrap CI, 0.0697–0.1433), an adjusted R^2^ of 0.0926 (95% CI, 0.0635–0.1376), and an RMSE of 10.078 ms (95% CI, 9.603–10.450). Confidence intervals for the remaining model specifications, individual partial R^2^ values and incremental contributions are presented in Table S6. General dominance analysis confirmed that FHR had the largest average contribution across predictor entry orders (Table S2). Its dominance weight was 0.05051, corresponding to 5.05 percentage points of total mPR variance and 51.23% of the variance explained by the full model. PVD accounted for 13.80% of explained variance, followed by BPD (10.51%), FOD (10.16%), GA (9.85%) and UA PI (4.44%). The unique contributions of GA, BPD and FOD were much smaller than their dominance weights, indicating substantial overlap in the information provided by these predictors.

Diagnostic testing indicated heteroskedasticity in the full model (Koenker–Breusch–Pagan *p* = 0.0184) and departures from residual normality in both models (Shapiro–Wilk *p* = 0.0279 and 0.0266). HC3 RESET tests did not detect the tested departures from linear functional form (*p* = 0.4001 and 0.3932), although these results do not establish correct model specification. Robust inference and exploratory influence analyses are reported in Table S5, with diagnostic plots in Figures S1 and S2. All 903 observations were retained in the primary analyses.

Thus, the full model including FHR, GA, BPD, FOD, PVD, and UA PI explained less than 10% of the total variability in mPR, whereas approximately 90% of the variance remained unexplained by the included covariates.

### 9.2. Robustness of Unexplained Variance Across Alternative Model Specifications

Numerical sensitivity-analysis results are provided in Table S3. The full OLS model had an apparent R^2^ of 0.0986 and a pooled out-of-fold R^2^ of 0.0826, with an out-of-fold RMSE of 10.167 ms. Excluding GA, log-transforming mPR or applying regularization produced similar validation performance. Gradient boosting achieved a higher apparent R^2^ of 0.1722 but a lower out-of-fold R^2^ of 0.0554 and an RMSE of 10.317 ms. Thus, the tested nonlinear specification did not improve internal validation performance over the linear models.

### 9.3. Exploratory Reduced Model

The exploratory reduced model containing FHR, PVD and UA PI retained similar apparent explanatory capacity to the full model (R^2^ = 0.0979 versus 0.0986, Table 4). FHR and PVD were associated with mPR. The incremental contribution of UA PI was small (ΔR^2^ = 0.00381), and its nominal significance depended on the covariance estimator: *p* = 0.0516 with conventional standard errors and *p* = 0.0450 with HC3 standard errors. This sensitivity warrants cautious interpretation and does not establish that UA PI is necessary for prediction (Tables S4 and S5). The two- and three-predictor models had nearly identical out-of-fold RMSE values of 10.137 and 10.132 ms, respectively (Tables S3 and S4). These results indicate that UA PI provided little incremental information and was not essential to the main finding.

Low VIF values for the predictors in the exploratory reduced model indicated less collinearity than in the full model.

### 9.4. Final Conclusions of the Analysis

In summary, sequential expansion of the models by additional predictor blocks resulted in only a modest increase in explained variance, whereas the dominant component of mPR variability remained unexplained by the included covariates.

The reduced model retained a similar proportion of explained variance while reducing predictor redundancy. These results describe the explanatory capacity of the included covariates and do not establish physiological independence of mPR.

## 10. Discussion

The present secondary analysis extends the previous publication by quantifying incremental contributions of predictor blocks, allocating shared explanatory information among correlated covariates, and comparing apparent fit with internal validation performance. The low overall explained variance was previously reported and is not a new finding [3]. The additional analyses showed that FHR had the largest average contribution across predictor entry orders, while biometric and Doppler blocks provided limited incremental information after FHR and GA.

The sensitivity analyses showed limited internal validation performance across the tested specifications. Although gradient boosting improved apparent fit, this improvement did not extend to held-out observations. The results therefore support limited predictive performance of the evaluated models within this cohort, but do not exclude unmeasured determinants or better performance from other model specifications.

The main clinical application of mPR in perinatology is the surveillance of fetuses whose mothers have anti-SSA/Ro and anti-SSB/La antibodies [1]. However, its potential utility may extend beyond this indication. Previous studies have explored the use of this parameter in pregnancy complications such as intrahepatic cholestasis of pregnancy [11] and in fetal channelopathies [12]. Direct assessment of fetal cardiac electrical activity is possible using non-invasive fetal electrocardiography (NI-fECG) and fetal magnetocardiography [2]; however, the use of these diagnostic methods in routine clinical practice remains limited because of restricted equipment availability.

The mPR parameter evaluated in the present study is a mechanical surrogate for atrioventricular conduction assessment. It does not directly measure fetal cardiac electrical activity, but rather its mechanical consequence, and therefore also incorporates an electromechanical delay component [13]. For this reason, mPR may differ from the true electrical AV conduction time and should be interpreted as an ultrasonographic correlate, rather than a direct equivalent of the PR interval on ECG.

In postnatal studies in children, age and sex have been shown to significantly influence conduction time, which has led to the development of sex- and age-specific percentile charts. The median PR interval increases from 108 ms in girls and 110 ms in boys on days 1–6 of life to 147 ms in females and 150 ms in males later in childhood and adolescence [14]. In prenatal studies, no sex-related differences have been demonstrated [15].

Similar to prenatal observations, the postnatal PR interval is associated with heart rate: it lengthens at lower heart rates and shortens at higher heart rates [16,17]. This indicates that the PR interval is not a fixed parameter, but rather a dynamic measure influenced by physiological state and autonomic modulation.

Some studies have even reported transient PR values ≥200 ms and first-degree atrioventricular block during rest or sleep [18]. Other publications indicate that variables such as body weight may also affect PR interval duration on electrocardiography. Sun et al. demonstrated that obesity is associated with a longer PR interval [19].

The variability of the PR interval assessed by postnatal electrocardiography depends on numerous physiological factors, some of which cannot be routinely evaluated in the fetus. One of the best-documented extracardiac factors associated with PR interval duration on electrocardiography is the concentration of free thyroid hormones [20]. Weaker associations have also been reported for race and parity in women [21,22].

Postnatal findings suggest that fetal mPR may also be influenced by physiological, technical and behavioral factors, although the present analysis cannot quantify their contributions.

In our analysis, FHR remained the strongest single covariate of mPR and retained an independent association with mPR after adjustment for the remaining variables included in the model. This is consistent with the physiology of the cardiac conduction system, particularly the atrioventricular node [23]. When added after FHR, GA increased R^2^ by 0.024 (*p* < 0.001). In contrast, the subsequent addition of BPD, FOD, and PVD produced a smaller increase (ΔR^2^ = 0.006; *p* = 0.112), and UA PI added a further 0.004 (*p* = 0.057). The latter two additions did not reach the conventional significance threshold in the nested-model comparisons. These findings indicate limited explanatory capacity of the included covariates within this cohort. The comparatively larger contribution of FHR suggests that heart rate merits consideration when mPR reference values are developed or interpreted. However, the association observed here is insufficient to define an FHR-adjusted reference range or to change clinical thresholds. The cohort comprised selected healthy fetuses, measurement repeatability was unavailable, and the models explained only a small proportion of observed mPR variance. Any proposed adjustment would require dedicated reference-range analyses and external validation.

The strong collinearity between GA and biometric parameters is not merely a statistical issue, but reflects the biological redundancy of several indices describing the same process of fetal maturation and growth. In the previous study, GA and EFW were excluded from the final model for this reason, namely because of multicollinearity [3]. The small sequential increments associated with biometric variables must be interpreted in relation to their entry after GA. General dominance analysis reduced dependence on this ordering by averaging contributions across all predictor entry orders. FHR retained the largest average contribution, whereas the information provided by GA and fetal biometry was largely overlapping. However, dominance analysis does not eliminate multicollinearity or establish distinct physiological effects. The additional scientific information therefore concerns the distribution and overlap of explanatory information and the performance of alternative specifications, rather than the previously reported low overall R^2^. General dominance analysis quantified FHR’s contribution across predictor entry orders, while hierarchical comparisons showed the limited incremental information provided by biometric and Doppler blocks after FHR and GA. Internal validation further distinguished apparent model fit from performance on held-out observations: the exploratory nonlinear model improved apparent fit but did not improve out-of-fold performance. Individual coefficients for highly correlated predictors in the full model should therefore not be interpreted as stable estimates of separate biological contributions.

The unexplained proportion of variance is a property of the fitted model in this sample, rather than a direct measure of conduction-system autonomy. Here, residual variability refers to variation in observed mPR values remaining after fitting a specified model, whereas unexplained variance expresses its proportion as 1 − R^2^. Neither quantity measures physiological independence. The unexplained variance may reflect unmeasured biological or behavioral factors, technical and measurement variability, restricted variation within a clinically selected population, and model misspecification. Restriction to fetuses with normal cardiac anatomy and function may reduce the range of both mPR and its covariates and thereby affect observed associations and R^2^. The present analysis cannot quantify the separate contributions of these sources or determine how much residual variance reflects intrinsic atrioventricular conduction properties.

The absence of repeated mPR measurements is particularly relevant to this variance-focused analysis. Without repeat measurements, we could not estimate within-fetus measurement variability or separate it from between-fetus biological variability. A potentially substantial, but unquantifiable, proportion of the residual variance may therefore reflect measurement error. Measurement error in the covariates may also affect the observed associations and model fit. Accordingly, the residual component cannot be interpreted as a quantitative estimate of biological variability in atrioventricular conduction.

The strengths of this study include the large size of the analyzed cohort, the homogeneous nature of the population comprising fetuses with normal cardiac anatomy and function, the use of a standardized mPR measurement protocol, and consistent acquisition by a single experienced fetal echocardiographer, although measurement repeatability was not assessed. Another important strength is the hypothesis-driven nature of this secondary analysis, which focused on the quantitative assessment of explained and unexplained variance rather than solely on statistical significance testing of individual predictors. In addition, the analysis included collinearity diagnostics, hierarchical modelling, sensitivity analyses, cross-validation, and evaluation of an exploratory reduced model with less predictor collinearity.

The models did not account for fetal sex, maternal age or BMI, diabetes, thyroid status, fetal behavioral state, examination quality or machine settings, because these variables were not available as covariates in the analytical dataset used for this study. Their omission may affect both the estimated associations and the proportion of variability explained by the models. Accordingly, residual variability cannot be attributed specifically to intrinsic conduction properties. Prospective studies incorporating these covariates and repeated mPR measurements are needed to distinguish additional biological contributions from measurement variability.

The cohort was recruited from a single referral clinic, which limits generalizability to other clinical settings and populations. Although gestational age ranged from 15 to 38 weeks, its distribution was concentrated around 25–29 weeks, as reflected by the interquartile range. The estimated associations and explained variance therefore primarily reflect the gestational-age distribution represented in this cohort and may differ at earlier or later stages of pregnancy. Moreover, restriction to healthy fetuses limits the range of physiological variation and may partly contribute to the low explained variance. External validation across centers and a more balanced gestational-age distribution is needed.

Additional limitations arise from the retrospective and secondary nature of the analysis. The study was based on the same source cohort that had previously been used to assess determinants of mPR; therefore, the present findings should be interpreted as complementary to the original publication. Complete-case analysis excluded 101 of 1004 records (10.1%) and may have introduced selection bias. Although available mPR and FHR measurements were similar between included and excluded records, the observed UA PI values differed. These comparisons were limited by variable-specific missingness, particularly because UA PI was available in only 27 excluded records. Reasons for missing measurements were not recorded in the analytical dataset; therefore, the missing-data mechanism could not be established, and bias related to complete-case selection cannot be excluded. Furthermore, the results were not compared with more direct methods of assessing fetal cardiac electrical activity, such as fetal magnetocardiography or non-invasive fetal ECG. The real-world nature of the cohort limited full control over measurement conditions, although the fact that all measurements were performed by a single experienced fetal echocardiographer increased the technical consistency of the data.

## 11. Conclusions

This secondary analysis extends the previous report by quantifying incremental and shared explanatory information and comparing apparent fit with internal validation performance. FHR accounted for the largest average contribution across predictor entry orders, while the additional biometric and Doppler blocks provided limited incremental information after FHR and GA. The evaluated alternative specifications did not substantially improve internal validation performance.

The low overall explained variance is consistent with the previous publication and is not a new finding. Model-unexplained variance cannot be attributed specifically to intrinsic atrioventricular conduction properties, because its biological and measurement-related components cannot be distinguished using the available data.

## Figures and Tables

**Table 1:** Baseline characteristics of the analytic sample (*n* = 903).

Variable	Value
Gestational age, weeks	26 (25–29)
Biparietal diameter (BPD), mm	68.4 (62.2–76.1)
Fronto-occipital diameter (FOD), mm	91.1 (83.2–100.7)
Fetal heart rate (FHR), bpm	143 (137–150)
Umbilical artery pulsatility index (UA PI)	1.08 (0.94–1.22)
Pulmonary valve diameter (PVD), mm	5.4 (4.8–6.2)
Mechanical PR interval (mPR), ms	116 (107–123)

Data are presented as median (interquartile range). Abbreviations: BPD, biparietal diameter; FOD, fronto-occipital diameter; FHR, fetal heart rate; UA PI, umbilical artery pulsatility index; PVD, pulmonary valve diameter; mPR, mechanical PR interval.

**Table 2:** Correlation structure and multicollinearity among candidate predictors (*n* = 903).

Panel A. Correlations of mPR with candidate predictors		
Variable	Spearman’s rho	*p*-value
Fetal heart rate (FHR)	−0.255	<0.001
Pulmonary valve diameter (PVD)	0.187	<0.001
Fronto-occipital diameter (FOD)	0.186	<0.001
Biparietal diameter (BPD)	0.186	<0.001
Gestational age (GA)	0.181	<0.001
Umbilical artery pulsatility index (UA PI)	−0.102	0.002
Panel B. Strongest pairwise correlations among candidate predictors		
Predictor pair	Spearman’s rho	*p*-value
GA vs. BPD	0.969	<0.001
GA vs. FOD	0.960	<0.001
BPD vs. FOD	0.954	<0.001
BPD vs. PVD	0.886	<0.001
GA vs. PVD	0.880	<0.001
FOD vs. PVD	0.869	<0.001
Panel C. Multicollinearity diagnostics		
Predictor	VIF	
Gestational age (GA)	19.94	
Biparietal diameter (BPD)	19.88	
Fronto-occipital diameter (FOD)	14.26	
Pulmonary valve diameter (PVD)	5.22	
Umbilical artery pulsatility index (UA PI)	1.20	
Fetal heart rate (FHR)	1.09	
Condition number	4.42 × 10^3^	

Panel A and Panel B values are Spearman correlation coefficients (rho); *p*-values are two-sided. Panel C reports variance inflation factors (VIFs) calculated from the multivariable predictor set. Higher VIF values indicate greater multicollinearity. Abbreviations: mPR, mechanical PR interval; GA, gestational age; BPD, biparietal diameter; FOD, fronto-occipital diameter; FHR, fetal heart rate; UA PI, umbilical artery pulsatility index; PVD, pulmonary valve diameter; VIF, variance inflation factor.

**Table 3:** Incremental explained variance across hierarchical models for fetal mPR (*n* = 903).

Model	Predictors Included	R^2^	Adjusted R^2^	ΔR^2^	RMSE (ms)	Unexplained Variance (%)	*p* for Model Change
Model 1	FHR	0.065	0.064	—	10.26	93.5	—
Model 2	FHR + GA	0.089	0.087	0.024	10.13	91.1	<0.001
Model 3	FHR + GA + BPD + FOD + PVD	0.095	0.090	0.006	10.10	90.5	0.112
Model 4	FHR + GA + BPD + FOD + PVD + UA PI	0.099	0.093	0.004	10.08	90.1	0.057

Models were fitted using ordinary least squares on the complete-case analytical sample. ΔR^2^ indicates the increase in explained variance relative to the preceding nested model. *p* for model change was derived from nested-model F-tests. Unexplained variance (%) was calculated as (1 − R^2^) × 100. Abbreviations: mPR, mechanical PR interval; FHR, fetal heart rate; GA, gestational age; BPD, biparietal diameter; FOD, fronto-occipital diameter; PVD, pulmonary valve diameter; UA PI, umbilical artery pulsatility index; RMSE, root mean squared error.

**Table 4:** Exploratory reduced model for fetal mPR with standardized coefficients (*n* = 903).

Model Summary						
Analytical sample (*n*)	903					
Model type	Ordinary least squares					
Predictors retained	FHR, PVD, UA PI					
R^2^	0.098					
Adjusted R^2^	0.095					
RMSE (ms)	10.08					
Predictor	Standardized β	95% CI lower	95% CI upper	Partial R^2^	*p*-value	VIF
FHR	−0.2292	−0.2936	−0.1648	0.0514	<0.001	1.07
PVD	0.1476	0.0786	0.2165	0.0193	<0.001	1.23
UA PI	−0.0671	−0.1346	0.0005	0.0042	0.052	1.18

The exploratory reduced model included predictors representing heart rate, cardiac size, and placental hemodynamics; UA PI was retained for domain representation despite its small incremental contribution. Standardized coefficients were obtained from an OLS model fitted after z-score standardization of both predictors and the outcome. Confidence intervals and *p*-values in this table use conventional OLS standard errors; HC3 robust inference is reported separately in Table S5. Partial R^2^ was calculated for each predictor from the corresponding t-statistic in the unstandardized OLS model. Abbreviations: mPR, mechanical PR interval; FHR, fetal heart rate; PVD, pulmonary valve diameter; UA PI, umbilical artery pulsatility index; VIF, variance inflation factor; CI, confidence interval; RMSE, root mean squared error.

## Data Availability

The dataset supporting this study has been deposited in a public repository and is accessible at the following URL: https://doi.org/10.17605/OSF.IO/E3XSH (accessed on 1 August 2025).

## Supplementary Materials

Supplementary Materials: The following supporting information can be downloaded at: https://www.mdpi.com/article/10.3390/biomedicines14102303/s1. Table S1: Comparison of the previous publication and the present secondary analysis; Table S2: General dominance weights and unique contributions to explained mPR variance (*n* = 903); Table S3: Sensitivity analyses and internal validation in 903 complete cases; Table S4: Reduced-model comparisons and LASSO retention; Table S5: Model diagnostics and robust inference; Table S6: Bootstrap confidence intervals for apparent model fit and contributions; Table S7: Missingness and comparison of included and excluded records; Figure S1: Diagnostics for the full six-predictor model; Figure S2: Diagnostics for the exploratory three-predictor model. Supplementary Methods describe cross-validation, preprocessing, model settings, performance metrics, model diagnostics, robust inference and bootstrap confidence intervals.
