# Supplementary material for: Explained and Residual Variability of the Fetal Mechanical PR Interval in Structurally Normal Hearts: A Secondary Analysis of Physiological, Biometric, and Doppler Covariates
Source: Biomedicines. 2026 Oct 10;14(10):2303. doi: 10.3390/biomedicines14102303 (PMC13701536; doi:10.3390/biomedicines14102303)
Supplement: Supplementary file 1 [file biomedicines-14-02303-s001.zip › biomedicines-4567397-supplementary.pdf]

Supplementary material for fetal mechanical PR interval analysis

Supplementary material for *Explained and Residual Variability of the Fetal Mechanical PR Interval in Structurally Normal Hearts: A Secondary Analysis of Physiological, Biometric, and Doppler Covariates*

Table S1. Comparison of the previous publication and the present secondary analysis.

Analytical component	Previous publication [3]	Present analysis
Primary objective	Identify physiological, biometric and Doppler correlates of mPR.	Assess allocation of explained variance and robustness across model specifications.
Data source and sample	925 fetuses reported in the publication.	1004 source records; 101 excluded for missing model variables; 903 complete cases. Reuse of source data, without an independent validation cohort.
Missing-data handling	Median imputation described for modelling.	Complete-case analysis using the same 903 records across the compared models.
Gestational-age comparisons	Kruskal–Wallis tests, Dunn post hoc comparisons and Jonckheere–Terpstra trend tests.	Not repeated.
Correlation analysis	Spearman correlations with mPR and comparisons of dependent correlations.	Correlations with mPR and among candidate predictors; contextual analyses rather than new associations.
OLS models	FHR–GA model and a multivariable model including FHR, BPD, FOD, PVD and UA PI.	Four nested models: FHR; addition of GA; addition of BPD, FOD and PVD; addition of UA PI.
Overall explained variance	$R^2 = 0.083$ for the FHR–GA model and approximately 0.099 for the final model.	$R^2 = 0.065$, 0.089, 0.095 and 0.099 across the four stages. Low overall R^2 is a previously reported finding.
Sequential model comparisons	No corresponding four-stage blockwise analysis reported.	$\Delta R^2 = 0.024$, 0.006 and 0.004; nested-model F-test p-values <0.001, 0.112 and 0.057, respectively.
Multicollinearity	Considered during variable selection; GA and EFW excluded from the final model.	Correlation matrix, VIFs and condition number; comparison with less redundant specifications.
Order-independent variance allocation	General dominance analysis not reported.	Added during revision: average incremental R^2 across all 720 predictor entry orders. FHR contributes 51.23% of full-model R^2.
Regularization and cross-validation	LASSO with five-fold cross-validation and a ranking based on standardized coefficients.	Regularized regression and ten-fold cross-validation described as robustness analyses. Reuse of these methods is not itself novel.
Additional sensitivity analyses	No corresponding log-transformation or gradient-boosting analyses reported.	Log-transformed mPR, GA-excluded model and exploratory gradient boosting described; quantitative reporting is addressed separately.
Reduced model	Five-predictor final model.	FHR, PVD and UA PI model: $R^2 = 0.098$ versus 0.099 for the full model; reduced predictor redundancy.
Interpretation and added contribution	Associations and limited overall explanatory capacity established.	More detailed variance allocation and model comparison; no independent validation or direct demonstration of physiological independence.

Notes. Participant counts are reported as stated in the previous publication and as verified in the dataset used for the present analysis. General dominance analysis was added during revision. Repeated associations and low overall R^2 are not presented as novel findings. Percentages of explained variance use full-model R^2 as the denominator.

Abbreviations: mPR, mechanical PR interval; FHR, fetal heart rate; GA, gestational age; BPD, biparietal diameter; FOD, fronto-occipital diameter; PVD, pulmonary valve diameter; UA PI, umbilical artery pulsatility index; EFW, estimated fetal weight; OLS, ordinary least squares; LASSO, least absolute shrinkage and selection operator; VIF, variance inflation factor.

Reference [3] (numbering follows the main manuscript): Swiercz G, Janiak K, Pawlik L, Mlodawska M, Kaczmarek P, Mlodawski J. Physiological Determinants of PR Interval in Healthy Fetuses: Insights from Correlation and Regression Modeling. J Clin Med. 2025;14:7522. doi:10.3390/jcm14217522.

Table S2. General dominance weights and unique contributions to explained mPR variance (n = 903).

Predictor	General dominance weight	Share of full-model R^2 (%)	Unique ΔR^2
FHR	0.050508	51.23	0.047835
PVD	0.013610	13.80	0.003495
BPD	0.010360	10.51	0.000337
FOD	0.010019	10.16	0.000074
GA	0.009712	9.85	0.000488
UA PI	0.004382	4.44	0.003668
**Total**	**0.098591**	**100.00**	—

General dominance weights represent average incremental R^2 across all predictor entry orders. Relative shares are expressed as percentages of the full-model R^2, not of total outcome variance. Unique ΔR^2 is the reduction in R^2 after removing the corresponding predictor from the full model and represents a squared semipartial correlation. General dominance weights allocate shared explanatory information and do not represent independent causal effects. Minor discrepancies in totals reflect rounding.

Table S3. Sensitivity analyses and internal validation in 903 complete cases.

Panel A. Apparent fit and pooled out-of-fold performance.

Model	Apparent R^2	Apparent RMSE (ms)	Pooled OOF R^2	Pooled OOF RMSE (ms)
Mean baseline	0.0000	10.615	-0.0027	10.629
Full OLS	0.0986	10.078	0.0826	10.167
OLS excluding GA	0.0981	10.081	0.0841	10.158
Log-mPR OLS	0.0981	10.081	0.0819	10.171
LASSO	0.0980	10.081	0.0855	10.151
Ridge	0.0973	10.085	0.0862	10.147
Gradient boosting	0.1722	9.657	0.0554	10.317
OLS FHR + PVD + UA PI	0.0979	10.082	0.0888	10.132
OLS FHR + PVD	0.0941	10.103	0.0880	10.137

Panel B. Variation across the ten outer test folds.

Model	Fold R^2, mean ± SD	Fold RMSE (ms), mean ± SD
Mean baseline	-0.0151 ± 0.0179	10.587 ± 0.962
Full OLS	0.0708 ± 0.0517	10.124 ± 0.951
OLS excluding GA	0.0724 ± 0.0515	10.115 ± 0.952
Log-mPR OLS	0.0699 ± 0.0524	10.128 ± 0.948
LASSO	0.0742 ± 0.0493	10.107 ± 0.962
Ridge	0.0748 ± 0.0471	10.104 ± 0.958
Gradient boosting	0.0447 ± 0.0483	10.270 ± 0.999
OLS FHR + PVD + UA PI	0.0770 ± 0.0522	10.090 ± 0.949
OLS FHR + PVD	0.0769 ± 0.0504	10.092 ± 0.969

Notes. Apparent metrics use predictions for the data used to fit the model. OOF metrics use one held-out prediction per record, pooled across ten folds. Mean fold R^2 need not equal pooled OOF R^2 because fold-specific outcome means and variances differ. SD describes variation across folds and is not a confidence interval.

All Panel A metrics are on the original mPR scale. Log-mPR predictions were retransformed using a training-set smearing factor. On the native natural-log scale, full-sample R^2 was 0.0985 and RMSE was 0.08724; these are not directly comparable with original-scale errors. The mean baseline predicts the training-set mean.

Table S4. Reduced-model comparisons and LASSO retention.

Panel A. OLS models fitted to the same 903 observations.

Predictors	R^2	Adjusted R^2	RMSE (ms)	AIC	BIC
FHR + PVD	0.09412	0.09211	10.103	6745.54	6759.96
FHR + PVD + UA PI	0.09794	0.09493	10.082	6743.74	6762.96
All six predictors	0.09859	0.09256	10.078	6749.08	6782.72

Panel B. Nested-model comparisons.

Comparison	ΔR^2	F statistic	p-value
Add UA PI to FHR + PVD	0.003811	3.7984	0.0516
Add GA, BPD and FOD to FHR + PVD + UA PI	0.000655	0.2171	0.8845

UA PI in the three-predictor model: unstandardized coefficient -3.269 ms per unit increase; 95% CI -6.561 to 0.023; $p = 0.0516$. The association does not meet the $p < 0.05$ threshold.

Panel C. LASSO coefficients and retention across outer training sets.

Predictor	Full-sample coefficient (ms per 1 SD)	Retained across outer training sets
FHR	-2.3464	10/10
GA	0.0000	1/10
BPD	0.2795	7/10
FOD	0.0093	3/10
PVD	1.2835	10/10
UA_PI	-0.6315	10/10

The full-sample LASSO penalty was $\alpha = 0.073564$. Predictors, but not mPR, were standardized. Coefficients are therefore not fully standardized beta coefficients. Retention means an absolute coefficient $>10^{-10}$. The full-sample LASSO retained FHR, BPD, FOD, PVD and UA PI; it did not select exactly the three-predictor OLS specification.

The two- and three-predictor OLS specifications have nearly identical out-of-fold RMSE (Table S3). These models were evaluated as fixed exploratory specifications. Their cross-validation does not independently validate the earlier choice of predictors using the same dataset. LASSO retention frequencies are descriptive, not significance tests or formal selection-stability estimates.

Supplementary methods for Tables S3 and S4

Analysis sample and predictor sets

The analyses used 903 complete records for mPR, FHR, GA, BPD, FOD, PVD and UA PI from 1004 source records. No imputation was applied. The full model included all six predictors. The GA-excluded model omitted GA only. Reduced OLS specifications contained FHR and PVD, with or without UA PI. The calculations reported in these tables were performed during revision with the settings below.

Cross-validation and preprocessing

The complete-case records retained their source order before shuffled ten-fold cross-validation (random_state = 20260924). The same outer splits were used for every model: three test folds contained 91 records and seven contained 90. Each record contributed one out-of-fold prediction. Splits were at the record level, treating each record as one fetus, as described in the study design. No stratification or grouping was applied. The seven-variable analysis file contained no identifiers permitting an independent check for repeated records from the same fetus.

For LASSO and ridge, predictor standardization was fitted within the training data through a pipeline. Hyperparameters were selected separately inside each outer training set using shuffled five-fold cross-validation (random_state = 20260925), minimizing mean squared error. Standardization was refitted within each inner training fold. The outcome remained in milliseconds. The apparent fits used the same inner tuning procedure on the complete sample. Selection of the original candidate variables was informed by earlier analysis of this dataset, so validation remains conditional on that candidate set.

Model settings

OLS included an intercept. The log model used the natural logarithm of mPR. Original-scale predictions were exp(predicted log mPR) multiplied by the mean exp(training log residual). The smearing factor was estimated separately in each outer training set.

LASSO searched 61 logarithmically spaced alpha values from 0.001 to 10, using max_iter = 100000 and tol = 10^{-8}. Ridge searched 41 logarithmically spaced alpha values from 0.0001 to 10000. The full-sample selected alpha values were 0.073564 for LASSO and 100 for ridge.

Exploratory gradient boosting used squared-error loss, 100 trees, learning_rate = 0.05, max_depth = 2, min_samples_leaf = 10, subsample = 1.0 and random_state = 20260924; remaining settings followed scikit-learn defaults. These settings were fixed before this validation run, without hyperparameter searching. This single specification does not exclude potential gains from other nonlinear models.

Performance metrics and model comparisons

R^2 was calculated as $1 - SSE/SST$. RMSE was the square root of the mean squared prediction error, with the number of evaluated records as denominator. Apparent and pooled OOF R^2 used the full-sample outcome mean in SST; fold R^2 used each test fold's outcome mean. Fold SD used the sample standard deviation (ddof = 1). Native log-scale fit is stated separately. Nested OLS comparisons used conventional F-tests. Confidence intervals for the UA PI coefficient used the conventional OLS covariance estimate and Student t distribution. These intervals do not incorporate model-selection uncertainty. AIC and BIC were calculated from the maximized Gaussian OLS likelihood, counting regression coefficients including the intercept.

Software: Python 3.12.14; NumPy 2.3.5; pandas 2.2.3; SciPy 1.17.0; scikit-learn 1.8.0.

Abbreviations: mPR, mechanical PR interval; FHR, fetal heart rate; GA, gestational age; BPD, biparietal diameter; FOD, fronto-occipital diameter; PVD, pulmonary valve diameter; UA PI, umbilical artery pulsatility index; OLS, ordinary least squares; OOF, out-of-fold; RMSE, root mean squared error; SD, standard deviation; LASSO, least absolute shrinkage and selection operator; AIC, Akaike information criterion; BIC, Bayesian information criterion.

These analyses extend Tables S3 and S4 using the same 903 complete cases. They quantify model diagnostics, sensitivity of inference and uncertainty in apparent model fit. Internal validation performance and its variation across folds are reported in Table S3.

Table S5. Model diagnostics and robust inference.

Panel A. Residual and influence diagnostics.

Diagnostic	Full model	FHR + PVD + UA PI
Koenker–Breusch–Pagan statistic	15.2535	2.0397
Koenker–Breusch–Pagan p-value	0.0184	0.5642
Ramsey RESET F statistic	0.9426	1.0363
Ramsey RESET p-value	0.3900	0.3552
HC3 RESET F statistic	0.9171	0.9343
HC3 RESET p-value	0.4001	0.3932
Shapiro–Wilk W	0.9962	0.9962
Shapiro–Wilk p-value	0.0279	0.0266
Maximum Cook's distance	0.0185	0.0173
Records with Cook's distance > 4/n	43	45
Maximum leverage	0.0898	0.0883
Records with leverage > 2p/n	69	63
Maximum |internally studentized residual|	2.9360	2.9283

The full model included FHR, GA, BPD, FOD, PVD and UA PI. Both models included an intercept. Here n = 903 and p counts coefficients including the intercept (7 and 4, respectively). The Cook's-distance threshold was 0.00443; leverage thresholds were 0.01550 and 0.00886. These are screening thresholds, not automatic exclusion criteria. All 903 records remained in the primary analyses.

The full-model heteroskedasticity test was significant, whereas the reduced-model test was not. Shapiro–Wilk tests detected departures from normality in both models. Neither conventional nor HC3 RESET tests detected the tested departures from linear functional form; this does not establish that the models are correctly specified. Diagnostic plots are provided in Figures S1 and S2.

Table S5 continued

Panel B. Coefficients with HC3 standard errors and 95% confidence intervals.

Full model

Term	Coefficient	HC3 SE	HC3 95% CI	HC3 p-value
Intercept	151.8836	7.5385	137.0885 to 166.6787	<0.0001
FHR	-0.2831	0.0403	-0.3622 to -0.2041	<0.0001
GA	-0.2889	0.4176	-1.1084 to 0.5306	0.4892
BPD	0.0854	0.1515	-0.2119 to 0.3827	0.5732
FOD	0.0265	0.1025	-0.1747 to 0.2278	0.7959
PVD	1.3103	0.7037	-0.0707 to 2.6913	0.0629
UA PI	-3.2278	1.6310	-6.4289 to -0.0267	0.0481

Exploratory reduced model

Term	Coefficient	HC3 SE	HC3 95% CI	HC3 p-value
Intercept	151.9024	7.0085	138.1475 to 165.6573	<0.0001
FHR	-0.2837	0.0401	-0.3624 to -0.2051	<0.0001
PVD	1.4319	0.3369	0.7707 to 2.0930	<0.0001
UA PI	-3.2693	1.6286	-6.4655 to -0.0730	0.0450

Coefficients are unstandardized: change in mPR (ms) per one unit of the predictor, holding other included predictors constant. HC3 changes standard errors and inference, but not OLS coefficients, fitted values or R^2. Confidence intervals and p-values use Student’s t distribution with 896 degrees of freedom for the full model and 899 for the reduced model.

UA PI in the reduced model: conventional 95% CI −6.5614 to 0.0229, $p = 0.0516$ (Table S4); HC3 95% CI −6.4655 to −0.0730, $p = 0.0450$. Its small contribution should be interpreted cautiously because its nominal significance depends on the covariance estimator. The HC3 analysis does not establish that UA PI is necessary for prediction.

Table S5 continued

Panel C. HC3 Wald F-tests for added predictor blocks.

Added block and comparison	F statistic	p-value
Add GA	22.1117	<0.0001
Add BPD, FOD and PVD	1.9736	0.1163
Add UA PI to five-predictor model	3.9164	0.0481
Add UA PI to FHR + PVD	4.0297	0.0450
Add GA, BPD and FOD to reduced model	0.2260	0.8783

The first three rows follow the hierarchical sequence FHR → FHR + GA → FHR + GA + BPD + FOD + PVD → full model. The final two compare reduced specifications. Tests evaluate whether coefficients of the added variables are jointly zero in the larger model. Numerator degrees of freedom are 1, 3, 1, 1 and 3; denominator degrees of freedom are 900, 897, 896, 899 and 896, respectively. They complement the conventional nested-model F-tests.

Panel D. Exploratory sensitivity to observations flagged by Cook's distance.

Model after omitting flagged records	n	R^2	Adjusted R^2	RMSE (ms)
Full model	860	0.1122	0.1059	9.247
FHR + PVD + UA PI	858	0.1208	0.1177	9.179

Records with Cook's distance > 4/903 in the respective original model were omitted only for this exploratory sensitivity analysis. Different records can be flagged by the two models. Selection on influence changes the analysis sample, so these metrics are not evidence of improved prediction and are not directly comparable with primary-sample or out-of-fold metrics. Primary analyses retain all 903 records.

The FHR coefficient remained negative (−0.2943 in the full model and −0.3124 in the reduced model); PVD remained positive (0.9497 and 1.3326); UA PI remained negative (−4.5096 and −4.1769). Limited explained variance persisted, with R^2 of 0.1122 and 0.1208 in these selected sensitivity subsets.

Table S6. Bootstrap confidence intervals for apparent model fit and contributions.

Panel A. Model fit: estimate (95% CI).

Model	R^2	Adjusted R^2	RMSE (ms)
FHR	0.0652 (0.0380 to 0.0996)	0.0642 (0.0369 to 0.0986)	10.263 (9.811 to 10.670)
FHR + GA	0.0889 (0.0571 to 0.1290)	0.0868 (0.0550 to 0.1270)	10.132 (9.675 to 10.526)
FHR + GA + BPD + FOD + PVD	0.0949 (0.0663 to 0.1382)	0.0899 (0.0611 to 0.1334)	10.098 (9.633 to 10.472)
Full model	0.0986 (0.0697 to 0.1433)	0.0926 (0.0635 to 0.1376)	10.078 (9.603 to 10.450)
FHR + PVD	0.0941 (0.0624 to 0.1339)	0.0921 (0.0604 to 0.1320)	10.103 (9.652 to 10.494)
FHR + PVD + UA PI	0.0979 (0.0660 to 0.1397)	0.0949 (0.0629 to 0.1368)	10.082 (9.623 to 10.469)

Panel B. Partial R^2 for individual predictors: estimate (95% CI).

Predictor	Full model	FHR + PVD + UA PI
FHR	0.05039 (0.02641 to 0.08196)	0.05145 (0.02704 to 0.08368)
GA	0.00054 (0.00000 to 0.00775)	Not included
BPD	0.00037 (0.00000 to 0.00741)	Not included
FOD	0.00008 (0.00000 to 0.00613)	Not included
PVD	0.00386 (0.00003 to 0.01641)	0.01927 (0.00561 to 0.04014)
UA PI	0.00405 (0.00004 to 0.01579)	0.00421 (0.00005 to 0.01616)

Panel C. Incremental R^2: estimate (95% CI).

Comparison	ΔR^2 (95% CI)
Add GA to FHR	0.02367 (0.00823 to 0.04678)
Add BPD, FOD and PVD after FHR + GA	0.00605 (0.00133 to 0.02218)
Add UA PI to the five-predictor model	0.00367 (0.00003 to 0.01427)
Add UA PI to FHR + PVD	0.00381 (0.00005 to 0.01449)
Add GA, BPD and FOD to FHR + PVD + UA PI	0.00066 (0.00027 to 0.01113)

Intervals describe uncertainty conditional on the specified models and available covariates. These are intervals for apparent fit, not for cross-validated performance. Full model: all six predictors. Percentile intervals for partial and incremental R^2 must not be used as significance tests, especially near zero; see the following page.

Supplementary methods for Tables S5 and S6

Model diagnostics and robust inference

OLS was fitted with an intercept. Residual-versus-fitted, normal Q–Q, scale–location and Cook's-distance plots were inspected for the full and three-predictor models. Internally studentized residuals were $e_i/[s\sqrt{(1 - h_i)}]$, where $s^2 = SSE/(n - p)$ and h_i is leverage. The Koenker version of the Breusch–Pagan test used n times the R^2 from regressing squared residuals on the model predictors and an intercept, with a chi-square reference distribution and degrees of freedom equal to the number of predictors. Shapiro–Wilk tests assessed residual normality.

Ramsey RESET added squared and cubed standardized fitted values to the original design and tested their joint contribution using a conventional F-test. An HC3 Wald F-test of the same two terms was also calculated. These tests target particular departures from the specified functional form and cannot rule out all nonlinearities or omitted variables. Leverage and Cook's distance were screened using 2p/n and 4/n, respectively; these thresholds did not define exclusions from the primary analysis.

HC3 covariance was calculated as $(X^TX)^{-1}X^T\mathrm{diag}[e_i^2/(1 - h_i)^2]X(X^TX)^{-1}$. Coefficient confidence intervals used the Student t critical value with $n - p$ degrees of freedom. Robust block comparisons used HC3 Wald statistics divided by the number of tested coefficients, with an approximate F reference distribution. HC3 inference accommodates heteroskedasticity but does not correct multicollinearity, omitted-variable bias, measurement error or model-selection uncertainty.

Bootstrap confidence intervals

Five thousand pairs-bootstrap samples of 903 complete records were drawn with replacement using NumPy default_rng(seed = 20260926). Each sampled row retained mPR and all six predictors together. The same sampled indices were used for every model and nested comparison within a replicate. Each model was refitted in every replicate; no model or variable selection was repeated. The procedure assumes independent records.

The 95% percentile bootstrap intervals were the 2.5th and 97.5th percentiles of the bootstrap estimates. These intervals describe resampling uncertainty and do not account for prior model selection or correct optimism in apparent performance. Coverage may be unreliable for contributions close to zero. In particular, sample partial R^2 and nested-model increments are nonnegative and usually positive even when population contributions are zero; a positive lower percentile limit must therefore not be interpreted as a significance test. Coefficient and block tests are reported separately in Tables S4 and S5.

$R^2 = 1 - SSE/SST$; adjusted $R^2 = 1 - (1 - R^2)(n - 1)/(n - p)$; $RMSE = \sqrt{(SSE/n)}$. Partial R^2 was (SSE without predictor − SSE with predictor)/SSE without predictor. Incremental R^2 was R^2 of the larger model minus R^2 of the smaller model. Partial and incremental R^2 therefore have different denominators and are not interchangeable.

Availability of additional covariates

The seven-variable analytical dataset contained GA, BPD, FOD, FHR, UA PI, PVD and mPR. Fetal sex, maternal age and BMI, diabetes, thyroid status, fetal behavioral state, examination quality and machine settings were not available as covariates in this dataset and were not included in these analyses. Their absence from this analytical file does not establish whether they were recorded elsewhere in the clinical source documentation.

Software: Python 3.12.14; NumPy 2.3.5; pandas 2.2.3; SciPy 1.17.0. OLS and covariance calculations used NumPy; distribution functions and Shapiro–Wilk tests used SciPy. Cross-validation settings and scikit-learn version are documented with Table S3.

Figure S1. Diagnostics for the full six-predictor model.

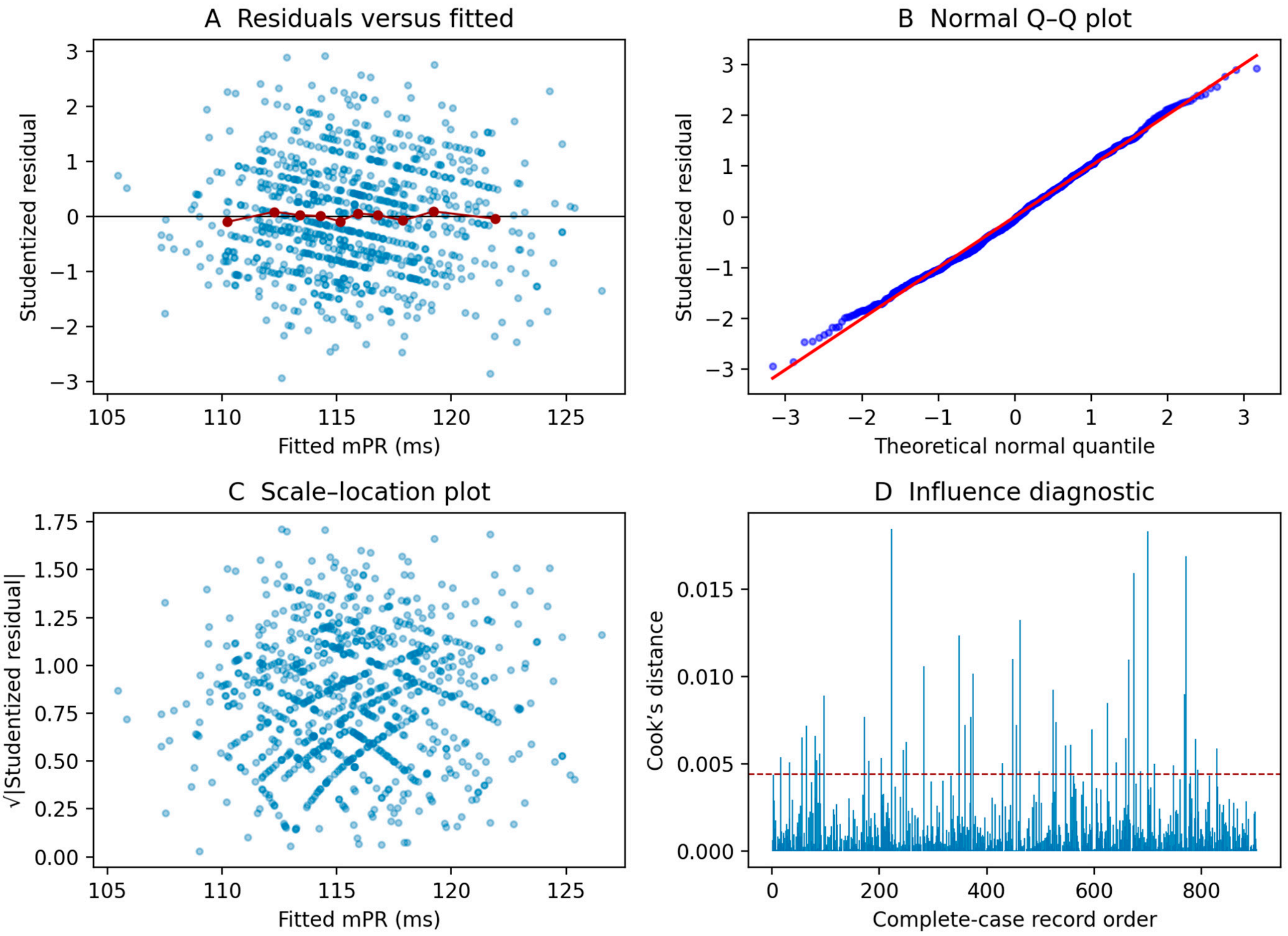

All 903 complete cases are shown. A: internally studentized residuals versus fitted mPR; the red line connects mean residuals within fitted-value deciles. B: normal Q–Q plot. C: square root of absolute internally studentized residuals versus fitted values. D: Cook's distance in source record order among complete cases; the dashed line indicates 4/903. Record order is not a temporal variable. Numeric tests and screening counts are reported in Table S5.

Abbreviations: mPR, mechanical PR interval; FHR, fetal heart rate; GA, gestational age; BPD, biparietal diameter; FOD, fronto-occipital diameter; PVD, pulmonary valve diameter; UA PI, umbilical artery pulsatility index; OLS, ordinary least squares; HC3, heteroskedasticity-consistent covariance estimator; CI, confidence interval; SE, standard error; RMSE, root mean squared error; SSE, residual sum of squares; SST, total sum of squares.

Figure S2. Diagnostics for the exploratory three-predictor model.

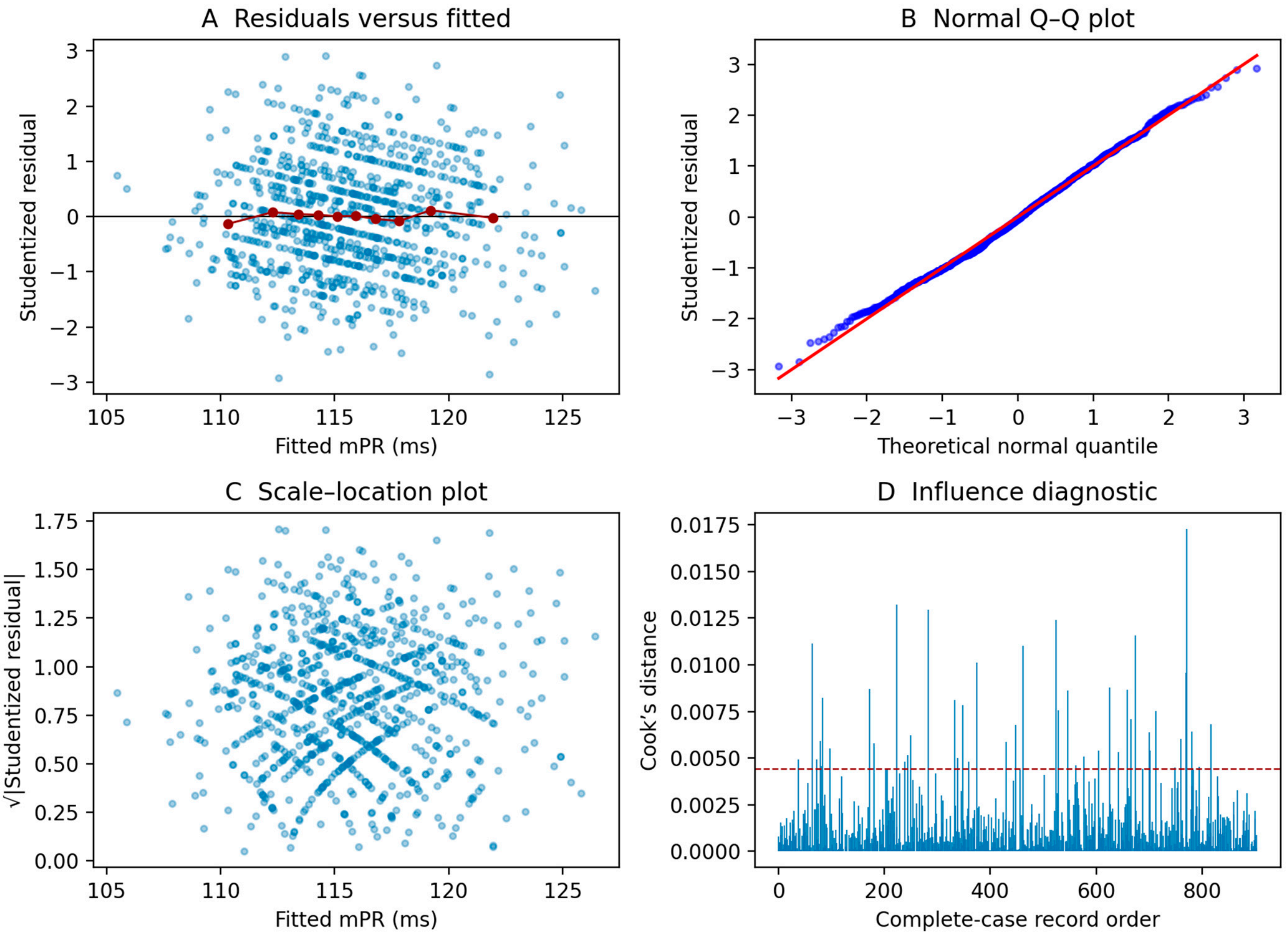

All 903 complete cases are shown. A: internally studentized residuals versus fitted mPR; the red line connects mean residuals within fitted-value deciles. B: normal Q–Q plot. C: square root of absolute internally studentized residuals versus fitted values. D: Cook's distance in source record order among complete cases; the dashed line indicates 4/903. Record order is not a temporal variable. Numeric tests and screening counts are reported in Table S5.

Abbreviations: mPR, mechanical PR interval; FHR, fetal heart rate; GA, gestational age; BPD, biparietal diameter; FOD, fronto-occipital diameter; PVD, pulmonary valve diameter; UA PI, umbilical artery pulsatility index; OLS, ordinary least squares; HC3, heteroskedasticity-consistent covariance estimator; CI, confidence interval; SE, standard error; RMSE, root mean squared error; SSE, residual sum of squares; SST, total sum of squares.

Table S7. Missingness and comparison of included and excluded records.

Of 1004 source records, 903 (89.9%) contained all seven required variables and 101 (10.1%) were excluded from complete-case modelling. No imputation was performed.

Panel A. Variable-specific missingness in the 1004 source records.

Variable	Missing, n	Missing, %
GA (weeks)	2	0.20
BPD (mm)	3	0.30
FOD (mm)	5	0.50
FHR (bpm)	17	1.69
UA PI	74	7.37
PVD (mm)	22	2.19
mPR (ms)	18	1.79

Counts overlap because a record may lack more than one variable. A total of 141 variable values were missing across 101 records.

Panel B. Mutually exclusive patterns among the 101 excluded records.

Missing variables	Records, n	% of excluded
UA PI	64	63.37
PVD	11	10.89
FHR, mPR	9	8.91
UA PI, PVD	4	3.96
FHR, UA PI, PVD, mPR	4	3.96
GA, BPD, FOD	2	1.98
FHR, PVD, mPR	1	0.99
PVD, mPR	1	0.99
BPD, FOD, FHR, PVD, mPR	1	0.99
FHR	1	0.99
FOD, UA PI	1	0.99
FOD, mPR	1	0.99
FHR, UA PI, mPR	1	0.99

Each excluded record contributes to one pattern only. Reasons for individual missing values were not encoded in the analytical dataset. The patterns cannot distinguish non-acquisition, technical failure or incomplete recording and do not establish the missing-data mechanism.

Table S7 continued

Panel C. Available measurements in included and excluded records.

Variable	Included (n = 903), mean ± SD	Excluded, available n	Excluded, mean ± SD	SMD
GA (weeks)	26.94 ± 3.63	99	26.14 ± 5.32	0.176
BPD (mm)	69.20 ± 10.17	98	66.77 ± 15.58	0.185
FOD (mm)	91.85 ± 13.00	96	88.92 ± 19.84	0.175
FHR (bpm)	142.98 ± 8.58	84	143.38 ± 8.98	-0.045
UA PI	1.08 ± 0.22	27	1.29 ± 0.47	-0.560
PVD (mm)	5.53 ± 1.09	79	5.51 ± 1.36	0.014
mPR (ms)	115.71 ± 10.62	83	115.64 ± 12.24	0.006

Panel D. Medians and interquartile ranges for the same available measurements.

Variable	Included, median (IQR)	Excluded, median (IQR)
GA (weeks)	26.00 (25.00–29.00)	27.00 (24.00–29.00)
BPD (mm)	68.40 (62.20–76.10)	68.60 (60.65–75.70)
FOD (mm)	91.10 (83.25–100.65)	90.25 (81.30–101.98)
FHR (bpm)	143.00 (137.00–150.00)	144.00 (137.75–150.00)
UA PI	1.08 (0.94–1.22)	1.20 (1.00–1.41)
PVD (mm)	5.40 (4.80–6.20)	5.60 (4.80–6.00)
mPR (ms)	116.00 (107.00–123.00)	116.00 (109.00–124.00)

Methods and interpretation

Each comparison uses all available values of that variable within the respective group. Included records have n = 903 for every variable; excluded-group denominators vary as shown in Panel C. Signed standardized mean differences (SMDs) were calculated as (mean included − mean excluded)/$\sqrt{[(SD^2 \text{ included} + SD^2 \text{ excluded})/2]}$, using sample SDs. Comparisons are descriptive, unadjusted and were not used as hypothesis tests.

Available mPR and FHR measurements were similar between groups. GA, BPD and FOD means were lower and their SDs larger among excluded records. Available UA PI was higher among excluded records (1.29 ± 0.47 versus 1.08 ± 0.22; SMD = −0.560), but it was observed in only 27 of 101 excluded records. This selected subset cannot represent the unobserved UA PI values in the other 74 records. The comparison cannot establish missing completely at random or exclude selection bias.

Abbreviations: GA, gestational age; BPD, biparietal diameter; FOD, fronto-occipital diameter; FHR, fetal heart rate; UA PI, umbilical artery pulsatility index; PVD, pulmonary valve diameter; mPR, mechanical PR interval; SD, standard deviation; IQR, interquartile range; SMD, standardized mean difference.
